# Smithsonian Institution

**Published:** 1851-07

**Authors:** 


### SMITHSONIAN INSTITUTION.

Our readers are apprised of the existence of an institution, at the city of Washington, founded by a foreigner, for the advancement and diffusion of knowledge among men. We have before us the fourth Annual Report of the Board of Regents of the institution, and we have read it with unfeigned pleasure. The establishment promises to confer incalculable benefits upon our country, by fostering and diffusing science and letters, and is therefore one concerning the organization, management, and success of which the members of the medical profession cannot feel indifferent. It is fortunate for the literary and scientific interests of our country that it has secured, as its head, a man so thoroughly prepared to conduct its affairs as Professor Henry.

The whole amount of the Smithsonian bequest received into the United States treasury was $515,169, and up to the 1st of July, 1846, the interest which had accrued on the same was $242,129, making in all $757,298. The first danger to which this bequest was exposed was, that it would be expended upon a costly edifice, the fate of so many similar bequests. The Regents very wisely determined to limit the whole expenditure on the building and the grounds to a sum not exceeding $250,000, leaving the principal intact; and in order that the accrued interest might not be exhausted it was resolved that this expenditure should not be made at once, but in the course of five years, and that in the mean time the sum of $242,000 should be invested so as to yield an interest which might in part serve to defray the expense of the building. The report goes on to state, that "after paying for more than one-half of the building, carrying on the operations of the institution, collecting a library and philosophical apparatus, the sum originally intrusted to the Regents has only been diminished by less than $16,000." So judiciously have the finances of the institution been managed that there is now every prospect, that at the end of the five years there will remain the sum of $150,000 in accrued interest to add to the principal.

Various schemes for appropriating the bequest have beeen proposed. Some were for collecting a magnificent library at Washington. Others were in favor of establishing a grand museum of objects of nature and art. A wiser system than either has been adopted, by which a great library will gradually grow up, extensive cabinets be formed, science be advanced as well as diffused, and in the mean time the principal of the donation remain undiminished. Too much credit cannot be given to the learned and able Secretary for the energy with which he has adhered to this plan of organization.

Scientific men are generally poor. They cannot often afford the expense of publishing the results of their labors. The Smithsonian Institution is ready to step forward and give them the necessary aid, one of its leading objects being the publication of memoirs consisting of positive additions to knowledge. We may mention, as an instance, that the late Dr. Troost, of Nashville, left an elaborate monograph on the *Crinoidea of Tennessee*, which the Institution has undertaken to publish, and which the lamented author could not have brought out in a style worthy of his subject. The service in this way rendered to science no one can calculate.

It is the aim of the institution to make a collection of books, pamphlets, &c., which shall become "a documentary history of American letters, science and art." The officers desire "that the collection shall be *complete without a single omission.*" They add, "we wish for every book, every pamphlet, every printed or engraved production, however apparently insignificant. Who can tell what may not be important in future centuries?" Y.

